# Implant Survival, Clinical Outcome and Complications of Megaprosthetic Reconstructions Following Sarcoma Resection

**DOI:** 10.3390/cancers14020351

**Published:** 2022-01-11

**Authors:** Christoph Theil, Jan Schwarze, Georg Gosheger, Burkhard Moellenbeck, Kristian Nikolaus Schneider, Niklas Deventer, Sebastian Klingebiel, George Grammatopoulos, Friedrich Boettner, Tom Schmidt-Braekling

**Affiliations:** 1 Department for General Orthopaedics and Tumor Orthopaedics, University Hospital Muenster, Albert-Schweitzer Campus 1, 48149 Muenster, Germany; christoph.theil@ukmuenster.de (C.T.); jan.schwarze@ukmuenster.de (J.S.); georg.gosheger@ukmuenster.de (G.G.); burkhard.moellenbeck@ukmuenster.de (B.M.); kristian.schneider@ukmuenster.de (K.N.S.); niklas.deventer@ukmuenster.de (N.D.); sebastian.klingebiel@ukmuenster.de (S.K.); 2 Division of Orthopaedic Surgery, The Ottawa Hospital, 501 Smyth Road, Ottawa, ON K1H 8L6, Canada; ggrammatopoulos@toh.ca; 3 Hospital for Special Surgery, 535 East 70th Street, New York, NY 10021, USA; boettnerf@hss.edu

**Keywords:** sarcoma, megaprosthetic, megaprosthesis, megaprostheses, tumor prostheses, tumour prosthesis, endoprosthesis, revision arthroplasty

## Abstract

**Simple Summary:**

Malignant bone and soft tissue tumors are usually surgically removed with an envelope of healthy tissue as a barrier. If located in the long bones of the upper and lower extremity, this approach leads to a large bone defect commonly affecting a joint. One way to rebuild the bone defect and the neighboring joint is the use of a megaprosthesis that is anchored in the remaining bone comparable to a conventional joint replacement. In general this approach is popular as it provides early stability and allows the affected patient to begin rehabilitation early on. However, complications leading to long-term unplanned reoperation are common. This article provides an overview of current implant survival, types of complication and long-term outcomes of megaprostheses used following tumor resection.

**Abstract:**

Megaprosthetic reconstruction of segmental bone defects following sarcoma resection is a frequently chosen surgical approach in orthopedic oncology. While the use of megaprostheses has gained popularity over the last decades and such implants are increasingly used for metastatic reconstructions and in non-tumor cases, there still is a high risk of long-term complications leading to revision surgery. This article investigates current implant survivorship, frequency and types of complications as well as functional outcomes of upper and lower limb megaprosthetic reconstructions.

## 1. Introduction

The treatment of extremity bone sarcoma is an interdisciplinary challenge. While (neo-) adjuvant treatment modalities vary among tumor histologies, study protocols and different oncological and patient factors, complete resection of the tumor is usually recommended across most tumor entities [1,2]. While historically, lacking efficient adjuvant therapies, a compartment or radical resection with amputation of the affected limb has been performed, a limb-sparing wide resection can be considered the standard approach in over 90% of bone sarcomas at present [3,4]. Following resection, surgeons face the challenge of reconstructing the bone defect and commonly the adjacent joint given the usual metaphyseal extent of bone sarcomas. In the last decades, megaprosthetic reconstruction using modern, modular endoprosthesis or tumor prosthesis has become a widely used, standard approach for the reconstruction of osteoarticular defects of the upper and lower extremity [4,5,6,7].

Megaprostheses have the advantage of a wide availability of different off-the-shelf modular implant systems that allow for individual, exact defect reconstruction, immediate, primary stability and the possibility to start early weight-bearing and functional rehabilitation. However, despite these advantages and all efforts to improve implant design, the massive implants are expected to experience long-term complications associated with revision surgery in almost 50% of reconstructions [4,8]. Furthermore, once a complication has occurred, patients are at risk for further revision surgeries and ultimately loss of the affected limb in cases of multiple revisions [9,10].

In recent years a comprehensive classification system was developed that allows for a clearer communication when reporting survival and failure of these implants. Henderson et al. [8] differentiate prosthetic failure that requires revision surgery into five subtypes: soft tissue failures (type 1), aseptic loosening (type 2), structural failure (type 3), infection (type 4) and local tumor progression or recurrence (type 5). However, it has been noted that there still are great differences in survival and incoherent reporting of failure in studies investigating megaprosthetic revision surgeries [11].

This review investigates current survivorship and complications of megaprosthetic reconstruction after upper and lower extremity sarcoma resection as well as present challenges and debates in this evolving field.

For this article the authors performed a selective literature review focusing on recent studies published after 2011 that applied the classification proposed by Henderson et al. [8]. Furthermore, reference lists were searched to identify additional articles. Generally, articles from large, dedicated centers in Europe and North America including a minimum number of patients or long-term studies were preferably included whenever possible.

## 2. Upper Extremity

The most common indications for modular megaprosthetic reconstruction after sarcoma resection involving the upper extremity are proximal humerus resections with or without involvement of the shoulder joint or scapula. In much rarer cases [8,12,13], total humerus resections, distal humerus resections, diaphyseal humerus resections (intercalary megaprosthetic reconstruction) and proximal ulna resection [13] can be addressed. In rare cases custom implants may be used for other forearm tumors [14] or after isolated scapulectomy [15], but there are no modular systems available for these indications to the authors’ knowledge.

### 2.1. Proximal Humerus Replacement (PHR)

Soft tissue failures (type 1) involving the surrounding tissues leading to dislocation, implant migration or complications regarding implant coverage and wound healing are particularly common in megaprosthetic reconstructions of the shoulder joint, which is best investigated for proximal humerus replacements (PHRs) [7,8,16,17]. The probability of soft tissue failure varies between studies. Henderson et al. [8] in their multi-institutional study including almost 350 prostheses found an overall rate of 4% revisions for soft tissue complications that however represent 25% of all revision of PHR performed; similarly, Bohler et al. [7] found a rate of 12% soft tissue complications that made up 50% of all complications in a study on 48 PHRs from a single center.

Despite these high rates of revision surgery for a soft tissue complication, there is some controversy with regard to when to perform revision surgery for the migrating or subluxated anatomical shoulder hemiprosthesis that is traditionally used. Cannon et al. [18] reported that more than 25% of anatomical hemiprostheses after proximal humerus tumor resection showed signs of proximal migration; however, in their cohort of 83 PHRs none were revised for that reason. Similarly, Nota et al. [19] have investigated the results of 84 proximal humerus megaprostheses and noted subluxation or migration in more than 40% of prostheses, but only performed revision in one case for that reason. Depending on the degree of resection the anatomical PHR hemiprosthesis is usually merely attached using soft tissue fixation to the remaining bone structures. We recommend conservative management as long as the patient is asymptomatic, unless perforation is imminent. Considering the fair results reported for this approach despite the high prevalence of radiological complications [18,19], aggressive surgical intervention does not seem warranted.

It appears logical that the likelihood for soft tissue failure is increased in resections that are more extensive. In particular, extra-articular shoulder resections involving the scapula (types IV–VI according to Malawer) [17,20] with megaprosthetic PHR have been shown to lead to high rates of prosthetic failure in more than 50% of cases. In particular, soft tissue revisions are common as 25% of patients required unplanned surgery for that indication according to a series of 55 procedures reported by Angelini et al. [17]. However, to our knowledge there are no studies that involve a control group comparing the results of intra- vs. extra-articular shoulder resections and subsequent megaprosthetic reconstruction.

One possible approach to reduce soft tissue complications is the use of synthetic mesh that is sutured on the body of the prosthesis and used to fixate the remaining muscle or capsule on the implant [21,22,23]. In particular, in extra-articular resections a megaprosthesis with a synthetic mesh can be used to attach the arm to the thorax as a “suspensionplasty”. While there is currently no robust evidence that this additional foreign material is associated with an increased probability of infection [22], surgeons should be aware of the fact that the increased surface area carries the risk of bacterial adhesion and removal of the mesh may be required in revision surgery in cases of infection. Furthermore, a modified acromion and musculus trapezius transfer in conjunction with megaprosthetic reconstruction has been described although the potential gain in function was limited and the infection rate was high [24].

One important innovation over the last 10–15 years has been the introduction of reverse total shoulder arthroplasty (rTSA) using a PHR in orthopedic oncology [25,26,27,28]. While usually an anatomic hemiprosthetic PHR has been used, reverse megaprosthetic TSA is an option when the axillary nerve, the deltoid muscle and the glenoid remain widely intact after tumor resection [25,26,27]. While these reconstructions can help in restoring function, with respect to soft tissue failures, “true” dislocations of the articulations can occur. Trovarelli et al. [27] report a dislocation rate of 18% in 22 patients treated with primary reverse TSA after tumor resection that all required prosthetic revision surgery and Streitbürger et al. [25] reported 22% dislocations in 18 patients with no revisions performed for this indication as damage to the axillary nerve or deltoid was preexisting or expected overall survival was deemed to be poor. Contrary to that, McLean et al. [28] reported no dislocations in eight reconstructions, which might be attributable to a different prosthetic design being used and higher degree of intact deltoid muscle and axillary nerve. Considering that all studies report short- to mid-term results with mean follow-up periods of around three years, long-term results are missing and will elucidate whether the complication rate is acceptable for the expected gain in shoulder function.

One of the most dreadful complications in megaprosthetic reconstructions is infection, which is reported to occur in around 5% of proximal humerus reconstructions [8], but can be much more common in total humerus replacement or extra-articular resections [17] where it has been reported in 12–19% of patients [8,16,19,29].

Although it is less common on the upper extremity compared to reconstructions around the knee [8], the treatment of megaprosthetic infection of humeral reconstructions can be associated with severe morbidity and multiple revision surgeries [19].

While it is generally accepted that infection is a common issue after megaprosthetic upper extremity reconstruction, even studies from large tertiary referral centers only report singular cases as part of published series [7,22,25,30,31] which makes it difficult to draw conclusions for general management. However, there is agreement that megaprosthetic infection should be managed similarly as conventional total joint arthroplasty infection and successful instances of component exchange, single-stage revision or two-stage revision have been reported [30,31,32,33]. Nonetheless, there appears to be a lack of studies investigating the outcome and management of upper extremity megaprosthetic infections, particularly considering the various factors that have been studied as predictors of outcome in non-megaprosthetic reconstructions such as microbiological findings [32]. Furthermore, despite its promising results in some lower-extremity reconstructions, the potential value of silver-coated implants has not been systematically studied on the upper extremity [34].

Aseptic complications (aseptic loosening, breakage of the implant or periprosthetic fractures) are considered to be a rarity for humeral reconstructions, largely because of the limited stress that is exhibited on an upper extremity compared to lower extremity prosthesis [19,33,35]. However, considering the expanding usage of reverse shoulder arthroplasties and improved patient survival as well as potentially increasing functional demand in long-term survivors [6], it is possible that glenoid loosening, as it has been reported for non-oncological shoulder arthroplasty [36], may also become more relevant in orthopedic oncology [28]. Furthermore, the radiological phenomenon of stress shielding around a humeral prosthetic stem has most recently also been reported for cemented and uncemented megaprostheses [37,38] and was found to be quite frequent. Nonetheless, in both studies, mechanical complications were rare and stress shielding might only play a role in individual cases.

Despite these complications, the overall implant survivorship has been reported to be rather good with Gosheger et al. reporting a five-year survivorship of 94% [4] and Bohler et al. a ten-year survival of 72% [7]. Additionally, around 85% long-term survival over more than ten years can be achieved based on the findings from a multicenter study [8].

The functional outcome of PHR greatly depends on the type of resection and prosthetic design used. While for extra-articular resections with suspension of the prosthesis on the remaining thorax or clavicle, hardly any active shoulder motion can be expected, but a functioning elbow and hand can usually be preserved, and intra-articular anatomic prosthesis can preserve some active motion [7,18]. For anatomic shoulder replacements, mean range of motion in abduction and forward flexion is usually limited to around 40–50° [18,39], but depending on the remaining deltoid, rotator cuff and ability to provide soft tissue attachment to the prosthesis, much better results are possible even with anatomic designs [23]. Tang et al. [23] have emphasized the role of soft tissue reconstruction using a synthetic mesh and compared active range of motion as well as Musculo-Skeletal Tumor Society [40] (MSTS) scores in 29 PHRs with or without mesh reconstruction. They found clear improvement of active motion and MSTS score when intra-articular resection had been performed and the deltoid function was intact. The MSTS scores for anatomical PHR have been reported to be between 20–25 points with lifting ability and hand positioning being the limiting factors [7,23]. Additionally to the use of synthetic mesh or during revision surgery, acromion and muscle transfers (modified by Gosheger [41]) can be an option to improve implant coverage and potentially regain function although there are no large-scale, long-term reports on this technique. However, pectoralis major or latissimus flaps might be required for additional implant coverage beyond any functional considerations.

Contrary to the rather limited function of anatomical PHR that was greatly dependent on the remaining soft tissue, reverse shoulder arthroplasty with a PHR has been reported to lead to reproducible, excellent function if the deltoid and axillary nerve can be preserved. Streitbüger et al. [25] reported a mean MSTS of 25 points and reliable restauration of anteversion and abduction to close to 90° if the deltoid can be preserved. Trovarelli et al. [27] even reported an MSTS of 29 points and mean abduction and anteversion of >100°. In our own practice, we recommend reverse designs for all patients in whom the deltoid and axillary nerve can be preserved and a glenoid component can be anchored in combination with meticulous soft tissue reconstruction using a trevira synthetic mesh.

### 2.2. Total Humerus Replacement (THR)

In rare cases of locally advanced tumors, skip metastasis, previous contaminating surgery or failed prior reconstructions, surgeons might use total humerus megaprosthetic replacement (THR) [29,42,43,44] in cases of insufficient bone stock for single joint replacements. There are only very few studies in the literature with Wafa et al. [29] reporting on 34 of such reconstruction, noting a higher failure rate compared to other humeral reconstructions but with good functional long-term outcomes, and concluding that THR is an appropriate extremity salvage treatment as the ten-year implant survival was 90% in their cohort. In particular, soft tissue complications and infection must be considered in THR as it follows the removal of the entire humerus and therefore most muscle attachments of the upper arm. Natarajan et al. [45] noted proximal migration throughout their series of 12 THRs, but only added a synthetic mesh to their reconstructive technique later in the series. Schneider et al. [44] investigated a cohort of 31 THRs performed for bone sarcomas and reported an implant survivorship of 74% after five years with infection and local recurrence being the main reasons for revision surgery. Patients with extra-articular resections were at increased risk for revision in this study.

Long-term functional outcome can be good to excellent [29,44] with an average MSTS of around 25 points and even the more demanding ASES (American Society of Shoulder and Elbow Surgery) score being good, with a median of 83 points [44]. However, considering the small number of patients available there is still uncertainty regarding what determines functional outcomes such as rTSA. Additionally, it is unclear if there is an effect on complication risk depending on adjuvant treatments such as radiation.

### 2.3. Distal Humerus Replacement (DHR)

The distal humerus is a rare location for bone tumors and therefore there is only limited data on megaprosthetic reconstruction available [8,12,46]. Megaprosthetic reconstruction of the elbow is considered a challenge due to the very limited soft tissue coverage available making the reconstruction prone to complications. Furthermore, functional outcome can be poor if the major nerves in the proximity are damaged during surgery or are resected due to tumor involvement. In recent years two studies have investigated modular megaprosthetic elbow reconstructions with Henrichs et al. [47] report on the outcome of twelve patients treated for malignant and recurrent locally aggressive bone and soft tissue tumors. A total of 25% of their patients underwent amputation for local recurrence and 25% had humeral stem loosening resulting in a revision-free implant survivorship of 64% at five years. Capanna et al. [48] reviewed 31 patients with modular elbow megaprostheses for bone and soft tissue malignancies. They noted a poor overall survival, but very good implant survivorship at five years of 93%. They reported one modular component failure and one infection. As the indications appear to be quite comparable, it is unclear why there are such stark differences in survival. Due to the rarity of studies published and rarity of elbow reconstructions performed using a megaprosthesis, only larger multicenter studies will be able to give a realistic implant survival estimate.

Regarding postoperative function, distal arm function is likely dependent on the ability to preserve the major nerves crossing the elbow region. Furthermore, an active extensor lag was noted in both studies. However, the MSTS scores published were fair in both studies at around 23/30.

A summary of complications and survival rates of “Upper Extremity” megaendoprostheses are shown in Table 1. 

## 3. Lower Extremity

### 3.1. Proximal Femoral Replacement (PFR)

The proximal femoral replacement (PFR) is one of the most frequently implanted megaendoprostheses and is used not only in tumor surgery but also increasingly in revision arthroplasty [49,50]. In particular, the proximal femur is a common site for bone sarcomas and metastases [51]. Instability (type 1 according to Henderson) is the most common complication after PFR and occurs in up to 1/3 of patients [52,53]. The loss of soft tissues and muscular attachments associated with oncological tumor resection is considered the main contributing factor for instability; however, advanced age, female gender and a primary bone tumor (as opposed to metastatic tumors) are also probably risk factors for dislocation. [52]. Dislocation usually occurs within the first months after surgery. In order to reduce the risk of instability, multiple efforts have been undertaken, with one study group in fact reporting no dislocations after PFR, by reconstructing the hip capsule using a synthetic device in which the abductor muscles and the iliopsoas muscle were reattached [4]. Furthermore, several studies recommend hemiarthroplasty instead of total hip arthroplasty reporting a vastly reduced risk of dislocation of up to 67%. There is a risk of long-term acetabular erosion by performing hemiarthroplasty; however, in these cases conversion to THA still can be performed successfully [52,54,55]. Therefore, a bipolar hemiarthroplasty PFR with meticulous soft tissue combination using a synthetic mesh appears to minimize the risk of instability and should be performed whenever possible [4,52]. It is unclear whether the use of dual-mobility cups, which have been shown to reduce the risk of instability in primary and revision THA, may also justify their routine use in PFR, although some reports with small numbers indicate that they might be an option if bipolar hemiarthroplasty is not possible. However, there are no long-term studies on the use of modern (dual mobility) acetabular components in PFR [56].

Aseptic loosening (Henderson type 2) is also a common failure mode in PFRs and may occur in 0–11% [57]. It is still debated whether cemented or uncemented coated stems provide the best long-term survival. Surgeons need to consider various factors such as the location with respect to the femoral isthmus, patient age, bone quality and associated adjuvant radiation or chemotherapy as they may all impair osseointegration. Generally, with modern implant designs, it appears that a stable long-term stem fixation can be feasible with both modes of fixation; however, porous stem collars or extracortical plates may provide for additional ingrowths and limit the potential impact of stress shielding osteolysis [58].

Although larger studies reported the structural failure rate (type 3) to be in the lower single digits (0–3%) [59,60,61], one study group reports the rates of mechanical failures to be as high as 19% [62]. In this specific study of 16 patients, one patient experienced a disconnection of a module and, at a later time, a cone loosening, and another patient experienced a screw fracture. This was partly explained with both patients being active in sports postoperatively. Nevertheless, no increased risk of structural failures in patients with a higher sports activity could be demonstrated [62]. However, with most implant designs that are available currently for PFR, structural failures are uncommon.

Even though infection rates of PFR are reported differently in the literature (6–19.5%) [4,49,59,63], the values are consistently much higher than for primary arthroplasties. The reasons for this are most likely rooted in the extent of resection with associated soft tissue damage, long operating times, large metal surfaces and the potential impact of adjuvant treatment, particularly radiation. Funovics et al. found a significant difference in periprosthetic infections between sarcoma (15.3%) and bone metastasis patients (1.1%) [49], but also the use of a PFR as a primary implant is associated with a significantly lower infection rate than when used in revisions (3.9% vs. 11.5%) [51].

A common approach to reduce the risk of infection is the use of coated implants. Currently, the most investigated coating is silver that has been applied by several manufacturers and has been shown to reduce the infection risk and the invasiveness of potential revision procedures (18.2% vs. 4.5%; *p* = 0.222), concluding that silver coating can reduce infection rates, without systemic side effects [63]. However, there are no randomized trials available and future studies should try to include other potential approaches such as the use of local or systemic antibiotics [49,64].

Despite these complications, numerous authors describe very promising limb salvage rates in oncologic patients, with values reported between 92% and 100% [4,49,54,55]. The survival rate of PFR at 5 years is reported to be between 55% and 90.7% [59,65,66,67,68], between 55 and 86% at 10 years [61,68,69] and 56% at 20 years [70].

### 3.2. Distal Femoral Replacement (DFR)

The distal femur is a frequent location for primary bone malignancies. At present, a limb-salvaging surgical approach can be chosen for most tumors and the use of a megaprosthetic distal femoral replacement (DFR) is a common reconstruction [71]. Nevertheless, the long-term failure rates of DFR are still quite high, although survival and common failure modes vary throughout different investigations.

The probability of a soft tissue complication (type I) after DFR was significantly higher for oncological indications than for revision arthroplasty (13.3% vs. 3.3%) [72]; these findings were associated with the considerably greater soft tissue trauma in the context of tumor resection, although adjuvant therapy certainly also played a role. However, soft tissue failure, i.e., tendon rupture, was less common, probably because patients are young and did not have previous surgical trauma.

According to Pala et al., soft tissue failure occurred in 7% of DFRs with a mean of one year, mostly involving superficial wound infections, hematomas or wound dehiscence [73]. These findings were confirmed by a recent review, which reported a mean soft tissue failure rate of 8.9% with a mean follow-up of 80 months in oncological indications [74]. Interestingly the authors reported on a higher type I complication rate in DFR, compared to PTR, which are often associated with a higher rate of complications.

Although the rate of aseptic loosening (type II) in oncology patients is lower compared to revision arthroplasty patients (17.8% vs. 23.3%) [72], aseptic loosening of the DFR is still one of the most common complications following bone tumor surgery. Henderson et al. reported an incidence of aseptic loosening to be highest of all locations on the distal femur (6.8%) [8]. which can probably be explained by the better bone quality in oncology patients, who are much younger on average. However, other studies reported much lower frequencies of this complication. In the review by Haijie et al. the rate was reported to be 8.8%, but without any significant difference in the choice of fixation (cemented vs. uncemented) or in the different mechanical prosthesis types (rotating-hinge vs. fixed-hinge) [74]. To avoid the complication of aseptic loosening, there are reports of an improved loosening rate with cementless HA-coated stems when compared to uncoated cementless stems (*p* = 0.06) [75]. The study group around Pala was also able to demonstrate this with HA-coated stems in the distal femur [73]. In case of the extensive resection of bone due to a tumor, the possibility of additional extra-cortical plate fixation may reduce the risk of aseptic loosening [76]. Furthermore, several manufacturers offer porous metal extracortical stem collars to avoid stress-shielding and loosening; however, there are no studies on this technique yet. The risk of type-II complications increased over time and greater resection length, younger age and smaller press-fit stems were risk factors for this specific complication [74].

The dynamic compression fixation was presented as an option to reduce osteolysis in the implantation of shorter stems and thus enable increased osseointegration, but aseptic loosening also occurred in 9.7% of cases, including periprosthetic fractures due to aseptic loosening in a study group of 82 patients [58]. However, inconsistent results can be found in the literature. The cementless stems are considered to have a better survival due to osseointegration, but the cemented stems show better stress-shielding properties [77]. Further long-term studies on the best stem fixation are certainly required in this context, especially for homogeneous study groups.

The rates of structural failures (type III) are very heterogeneous. Some studies did not report any structural failures [73], a femoral stem breakage rate of 2.8% [4] and complications with the rotating hinge mechanisms in 11.8% of cases, after a mean time of 159 months [71]. In contrast, structural failures were the most common complication in other study groups (37.3%) [72]. In the case of bushing wear, there is no consensus as to whether this is considered a structural failure or maintenance. Some authors see this complication as normal wear and do not classify it as a complication [5]. In our department, bushing wear is considered a complication because we found an increased rate of infection after the replacement of the bushings. Our own study group was able to show that the risk for a second revision increases after the first revision [9]. Again, these problems occurred more frequently in oncologic than in revision arthroplasty patients (40% vs. 33%), which we relate to the significantly lower age of the oncological patients (46 years vs. 71 years) and the consequently increased activity level [72]. Gender did not have any effect on the complication rate or survival of the prostheses, but patients who were over 60 years of age when the prosthesis was implanted were significantly less likely to experience mechanical problems or to require revision of the prosthesis [78]. Although implant breakage occurred more frequently with the rotating-hinge prostheses, they required revision less frequently because of the bushings. The authors concluded that modern implants, particularly modern polyethylenes, would decrease revision rates of DFR in the future [78]. Similarly, in a recent review of DFR in oncologic diseases, structural failures were the most common complications, consisting of bushing wear (13%) and implant breakage (4.6%) [74]. The authors reported bushing wear being significantly more common in cemented than in cementless prostheses (18.3% vs. 10.4%; *p* ≤ 0.001). In contrast, broken implants were significantly more common in cementless prostheses (5.7% vs. 3.5%; *p* = 0.012) and rotating-hinge prostheses (4.6% vs. 3.1%; *p* = 0.046). These results may be surprising, since the mechanical stress on the implants should be reduced structurally, especially due to the possibility of greater freedom of movement, and thus the risk of implant fracture or aseptic loosening should be reduced [78,79,80]. On the contrary, it could be shown that bushing wear was reduced by the rotating-hinge prostheses, which in turn could indicate a reduced mechanical stress. Lately there have been rising concerns regarding metal-on-metal (MoM) bushings and the subsequent increase in serum metal ion levels, which in one study were elevated in 70% of patients with MoM hinged revision knee prostheses [81]. In the future, there will be a need for larger studies that closely monitor the metal ions of these specific patients and could determine a valid threshold. It remains unclear which prosthesis types or fixations should be used to decrease the structural failure rate in the future.

Infection (type IV) is described in many studies as the most common problem after DFR, and always represents a serious complication, resulting in necessary amputation in 4.5% of patients [78]. Nonetheless, being the most common complication (8.5%), the infection rate in oncologic patients was significantly higher, compared with revision arthroplasty patients (11.4% vs. 4.1%), which is related to chemotherapy and the subsequent immunosuppression as well as the significantly higher intraoperative trauma due to the resection of bone and soft tissues in the primary surgery. Henderson et al. similarly reported the risk of infection of DFR as 8.3% [8] and Mavrogenis et al. as 8.2% [82]. Likewise, the overall infection rate of DFR in tumor cases was indicated by Haijie et al. to be 8.5%, with significantly higher incidence of infection in cementless implants, compared to cemented (9.0% vs. 5.8%; *p* = 0.003) and rotating-hinge prosthesis in comparison to fixed-hinge implants (10.2% vs. 4.4%; *p* ≤ 0.001) [74]. However, resection height also appears to have a significant impact on postoperative infection risk, with the extraarticular resection in the distal femur being associated with a 6.2-fold increased risk of infection compared with intraarticular resection (*p* = 0.004) [4].

Pala et al. reported a failure rate in this type of megaendoprosthesis of 26.7% and a mean time to failure of 3 years in their cohort of 187 DFRs—infections being the most frequent type of failure (8.5%), followed by soft tissue failures (7%) and aseptic loosening (5.3%). Interestingly, no structural failure, including bushing wear, occurred in this population [73]. In contrast, a review of 33 studies found the most frequent complication to be the bushing wear (13%), followed by soft tissue failure (8.9%), aseptic loosening (8.8%) and infection (8.5%) [74].

A recent oncological review reported the 5-, 10-, 15-, 20- and 25-year survival of DFRs to be 78%, 70%, 61%, 38% and 36%, respectively [74].

### 3.3. Proximal Tibial Replacement (PTR)

Although 15% of all osteosarcomas are found in the proximal tibia, surgical treatment continues to present difficulties to the surgeon [83], particularly soft tissue coverage can be an issue. Numerous studies have reported the failure rate in the proximal tibia to be the highest in the entire lower extremity [73,84,85], with some authors reporting it as high as 56% [86]. Pala et al. reported an overall failure rate of 36.7% in their collective in PTR, and again the largest group (13.3%) consisted of soft tissue problems, followed by infections as the second most common complication with 11.6% [73]. Mavrogenis also reported an overall complication rate of 25%, albeit infection (12%) was the most common, followed by aseptic loosening (6%) [87].

Soft tissue failures (type I) are common in PTR and were reported to be as common as 13.3% [73]. This is firstly related to the difficult soft-tissue coverage in the proximal tibial region and secondly due to the need to reconstruct the extensor mechanism as it is essential for knee functionality [65]. During resection of the proximal tibia, the extensor mechanism must be detached and adequately readapted to the megaendoprosthesis. To complicate matters, even without the need for tumor resection there are few soft tissue reserves, one the proximal tibia and the anterior tibial edge [83,88,89]. In particular, the risk of postoperative wound healing problems was significantly increased in patients with a high BMI or after previous radiotherapy [88]. Unfortunately, however, it is precisely these postoperative wound-healing disorders that pose a great risk of developing a periprosthetic infection in the further course; in some studies, 35–44% of patients with a postoperative wound-healing disorder developed a periprosthetic infection in the area of the proximal tibia in the subsequent period [86,88]. Thus, it is considered a high priority to provide adequate soft tissue coverage after the implantation of a PTR [88] and for this reason, some surgeons recommend the routine use of a gastrocnemius muscle flap [80,83,88,90]. According to a study by Hardes et al. (*n* = 98), soft-tissue failures occurred in 17.3% of patients after implantation of a proximal tibia due to oncologic diagnoses [89], while other studies report a tissue failure rate of 10% [86]. In contrast, however, a recent review consisting of 15 studies reported only 5.1% soft tissue failures in PTR, highly significantly lower even than in DFR (8.9%; *p* = 0.001) [74]. Thus, the literature remains highly discordant here as well and there is definitely a need for more extensive research in this field.

Although the literature reports relatively similar results regarding the aseptic loosening rates (type II) in DFR from 7.2% to 12%, the 5-year survival rate regarding stem fixation (cemented vs. cementless) was not different and no other significant causes could be found [74,86,88].

The structural failure rate was higher, particularly due to the necessary replacement of bushings, which were required in 8.6% to 19% of the patients, after a median of 69 months [74,80,86] with this explained by the increased torsional and shear forces that occur with the bushings. The risk is significantly higher with fixed-hinge implants than with rotating-hinge prostheses [74].

In comparison with other lower extremity megaendoprostheses, Mavrogenis et al. reported a higher infection rate of PTR (11.9%), in comparison with DFR (8.2%), PFR (6.5%) or even TFR with only 3.4% [82]. Other studies reported even higher values of 15–16.8%, with a cumulative incidence of 7% at one year, 17% at five years, and 25% at 10 years [4,86,88], with Staph. epidermidis being the most common pathogen [88]. Interestingly, fewer infections occurred in the silver-coated PTR group than in patients in the titanium group (12.5% vs. 19%), and silver coating was associated with a hazard ratio of 0.79 [89]. Similarly, a better outcome of silver-coated implants in the treatment of periprosthetic infections in megaprostheses was reported [66], with a re-infection rate of 10.3% in the silver-coated group and 17.5% in the non-silver group (*p* = 0.104) during a two-stage exchange in DFR and PTR. In addition, in the case of re-infection, amputation was significantly less often required in patients in the silver group, compared to the non-silver group (33% vs. 80%; *p* = 0.047). Larger studies would certainly have to clarify the significance of the silver coating; the silver coating can probably only prevent or reduce biofilm formation, but not infection of the periprosthetic tissue [34]. Furthermore, many complications that have been extensively investigated in arthroplasty, such as the type of microbial detection and the duration and type of antibiotic treatment, remain inadequately investigated in tumor collectives.

Secondary amputation had to be performed in 8.2% of patients, and the survival rate of the PTR was reported at 94.9% after one year, 90.5% at two, 79.2% at five and 74.5% at ten years [89]. These data are similar to a review that reported a survival rate at five years of 75%. However, after five years there was a significant decrease in survival from 60% at 10 years, 55.3% at 15 years and only 25.1% at 20 years [74]. Comparing the survival of megaprostheses at different sites of the lower extremity, a reduced survival of the PTR was shown in contrast to the femoral implants, which was also explained by the problems in soft tissue coverage [86].

### 3.4. Total Femoral Replacement (TFR)

TFR is an option in locally advanced tumors or in the context of prior contaminating surgery, skip lesions or in pathological fractures if the entire femur needs to be resected. Furthermore, it can be used in megaprosthetic revision surgery if severe bone loss is present, and salvage of the hip or knee joint is considered impossible. However, the expected risk of complications and revision surgeries is high. Nonetheless, considering that for many patients with extensive tumors amputation as a hip disarticulation must be considered alternatively, TFR is a reasonable option for a limb-saving approach.

Due to the extent of soft tissue dissection and loss of muscle attachments, soft tissue failures (Henderson type 1), including hip instability, are common. Toepfer et al. reported soft tissue failure in six of nine cases (67%) in the oncology group, of which dislocation occurred in three patients (30%) [67]. Sevelda et al. found a soft tissue failure following tumor resection in 13 patients (38%), including 8 patients with at least one dislocation (23.5%) [91]. Although soft tissue failures were less frequent in a study by Medellin, dislocation still occurred in 10% of their patients [71]. Considering the retrospective nature of the aforementioned studies and the small numbers gathered over long periods of time, one potential reason for hip instability is the type of acetabular reconstruction. In the dual mobility cup group, no dislocation occurred, but in contrast, cemented acetabular cups had the highest dislocation rate in this study group (26%), followed by bipolar heads with a dislocation rate of 14%. In conclusion, the authors recommend the use of dual mobility cups in combination with TFR.

In order to reduce the dislocation rates with these implants at risk, Du et al. attached the muscles to the tumor prosthesis with a strip of industrial-strength polyester fiber [92]. No postoperative dislocation occurred in the 12 TFRs treated with the synthetic device, but 12 dislocations (26.1%) occurred in the control group (*n* = 46). Although the infection rate of synthetic devices is controversial, the rate in this study group was lower with the synthetic device, compared to the control group (8.3% vs. 10.9%, *p* = 0.797). Surgeons should therefore perform meticulous soft tissue reconstruction using a synthetic attachment tube whenever possible. However, future studies are needed to evaluate the use of different acetabular components and synthetic soft tissue attachment evaluating potential adjuvant treatments and long-term survival.

Due to the replacement of the entire femur, aseptic loosening can only occur in the tibia or, if THA with a cup is performed, at the hip. Several authors report no aseptic loosening or a low failure rate due to type 2 failures [8,67,91]. However, Medellin et al. found aseptic loosening in 3 of their 81 patients (3.7%), in long-term survivors after 129–159 months [71].

While the rate of structural failures is not considered high despite the size of the reconstruction, [8], there still is the risk of bushing wear at the knee joint. Toepfer et al. reported bushing failure in 2 of 22 patients (9%), although they were able to perform uncomplicated bushing exchange [67]. Medellin et al. found structural failure in seven patients (8.6%), with complications occurring less frequently in TFRs with rotating hinge knees than in those with fixed hinge knees [71].

Infection is a devastating complication after TFR and accounts for 42.9% of all complications in TFRs [8]. In the retrospective study by Medellin, postoperative infection occurred in 18.5% of 81 patients and there was a threefold risk for infection if TFR was performed as a revision case (8% vs. 25%; *p* = 0.001) [71]. In their study, with the numbers available, silver coating did not reduce the risk for infection (17% vs. 19%; *p* = 0.869) and 9% of patients still underwent hip disarticulation to eradicate the infection. However, as there are different types of silver coating, future studies should be performed as silver has been shown to reduce the infection risk in other high-risk situations. Additionally, the surgical and antibiotic treatment of infected megaprostheses is debated; therefore, future studies must consider multiple aspects such as microbiology in treating these patients.

The revision free survival rate after 5 years was 71% and was still 63.3% at 10 years, and especially when using the TFR as primary implant a good survival rate could be achieved [71]. The limb survival time was 62 months, 13.5% of patients requiring amputation during follow-up. Although Sevelda reported a relatively high complication rate, an excellent implant survival of 97% at 5 years was still reached [91]. To further improve survival, the authors recommended the use of a dual mobility cup in the acetabulum and a rotating-hinge prosthesis in the knee joint to reduce the risks of mechanical or soft tissue failure [71]. Nonetheless, infection remains a major problem.

### 3.5. Combined Distal Femoral and Proximal Tibial Replacement (CFTR)

Although the most common locations of sarcomas are in the distal femur and proximal tibia [93], there is little data on the combination of these two megaprosthesis types [94]. However, according to Henderson, this combination had the highest failure rate and poorest survival of all tumor prostheses [8]. Sevelda et al. followed up 39 of these prosthesis combinations, with 37 patients operated on for sarcoma and 2 for metastases [94]. Overall revision-free survival was 3.7 years, but with a significant difference (*p* = 0.02) between primary surgeries (6.1 years) and revision surgeries (1.2 years). The most common types of implant failure were infections (16/39) and soft tissue failures (12/39). Of the 16 infections, 8 were cured, but 5 patients developed chronic infection and 3 patients required amputation. In the soft tissue group, eight patients developed extensor mechanism insufficiency. In this context, the use of synthetic devices to bridge or reinforce ligamentous structures, especially to reattach the extensor mechanism in the knee, is discussed controversially. Based on the study data, reconstruction with a synthetic device has a trend toward inferior survival and the use of the gastrocnemius muscle flap may be the more successful method. This is currently discussed controversially in other studies; some report an increased risk of amputation after combining a synthetic device and a tumor prosthesis in the knee joint [95], but others find no increased infection rates with this combination in the proximal tibia [96]. The third most common failure type was structural failure (9/39), including five prosthesis fractures. The authors explained this fact by the large leverage effect of this type of prosthesis. Nevertheless, aseptic loosening occurred in only one case. In terms of functional outcome, the mean MSTS scores were better with primary implantation, compared to the use of the CFTR as a revision implant (83% vs. 70%; *p* = 0.041). Based on the 94% limb survival rate, the authors recommended the use of this prosthesis, despite the frequent need for revision surgery, especially in early years.

A summary of complications and survival rates of “Lower Extremity” megaendoprostheses are shown in Table 2.

## 4. Functional Outcome

The best functional outcomes in adults with a megaendoprostheses for osteosarcoma were reported to be the DFR with a mean MSTS score of 85%, followed by the PFR (81%), the PTR (75%) and the TFR (71%) [65].

Hobusch et al. found 80% of the patients after implantation of a PFR due to a sarcoma were able to return to low-impact sports in the postoperative course, even similar to patients after primary total hip arthroplasty (THA) [62]. Although recovery in the sarcoma patients took longer, this could be explained by the significantly greater soft tissue trauma associated with the oncologic resections, compared with primary THA.

More than half of the patients achieved an MSTS score above 90% postoperatively after implantation of a DFR, which corresponds to a functionally near-normal value [65]. This not only allowed patients to return to daily life in many cases but could reduce the mental stress of this disease [65]. Similar functional results were also obtained by Schwartz et al. who reported a mean MSTS score of 86.7% and even a postoperative mean knee flexion of 110° in DFR [97].

Ruggieri et al. reported on 15 patients after implantation of a TFR assessed with the MSTS score. The mean score was 66% and the active ROM in the knee was 60° [55]. All patients were able to stand and walk short distances. Du et al. reported a postoperatively better range of motion, a highly significantly better HHS score and an increased MSTS score (70.4 vs. 80%, *p* ≤ 0.001) in patients treated with a synthetic device in the hip region during implantation of the TFR [92]. Compared to PFRs or DFRs, there was a significantly worse functional outcome after implantation of a TFR; however, the authors noted that functional scores were even worse after hip disarticulation [98].

Goryn et al. explained the poorer function of proximal tibial replacement in their study population by the need for detachment of the patellar ligament and the consequent significant weakening of the extensor apparatus, which is important for knee functionality [65]. Nevertheless, very good results were shown in both DFR and PTR, with excellent results in 80.2% and good results in 16.6%, with no significant difference between the two types of prostheses (*p* = 0.306) [73]. Mavrogenis et al. reported a mean MSTS score of 77% after PTR, showing a significant better score with the rotating-hinge prostheses, in contrast to the fixed-hinge implants (*p* = 0.042) [87], with further successive improvement of the functional scores in the 10 years after surgery. Encouragingly, patients after tumor resection and endoprosthetic reconstruction of the lower extremity demonstrated similarly high activity compared with patients after a primary hip prosthesis, although patients after implantation of a proximal tibia had decreased strength in knee flexion and extension [99].

The literature is conflicted on the issue of the best possible level of loading, in daily life and in sports, so there is currently no clear guideline for surgeons on what to advise their patients regarding postoperative exercise. In particular, the impact of postoperative loading on long-term survival of megaendoprostheses remains unclear. Although Ollivier et al. reported that higher activity levels led to risk of implant failure and lower implant survivorship [100], this could not be demonstrated in another study [62]. This crucial question will hopefully be further clarified in larger studies in the future, since exercise and return to activities of daily living also represent a high level of quality of life. This is especially relevant for the young oncology patients, for whom it is also psychologically of utmost importance not to be stigmatized by the disease. The study of Bernthal gives a glimmer of hope for these oncological patients due to its good postoperative results, comparable to a primary THA [99].

Thus, the question of the best prosthesis type and the most sustainable fixation in terms of aseptic loosening remains unanswered.

## 5. Conclusions

Due to the expanded use of megaprosthetic reconstructions following sarcoma resection, but also in patients with metastatic bone disease or in non-tumor revision arthroplasty cases it remains important to investigate and report implant survival and revision surgeries. While improvements in implant design may have improved mechanical complications such as breakage, wear or loosening, periprosthetic infection and repeat revision surgeries present an increasing and serious challenge. These future challenges must be addressed by researchers employing uniform definitions and classifications that, particularly for infections, have only recently been implemented by the revision arthroplasty community. Furthermore, modern statistical analysis such as competing risk analysis might offer a more realistic methodological approach to correctly estimate implant survivorship. Further long-term studies regarding the optimal management of complications are needed as well as studies on functional outcome and quality of life, especially in long-term surviving patients.

## Figures and Tables

**Table 1:** Summary of survival and complications “Upper Extremity”.

	Survival	Complications			
	(60 months)	Soft tissue failures (type 1)	Aseptic loosening (type 2)	Structural failure (type 3)	Infection (type 4)
Proximal Humerus Replacement (PHR)	90–94% [4,8]	4–50% [8,17,27]	0–2% [8,19,38]	0–1% [8,19,38]	5–19% [7,8,17]
Total Humerus Replacement (THR)	74–90% [29,44]	3–6% [29,44]	0% [8,44]	0–9% [8,29,44]	14–19% [8,29,44]
Distal Humerus Replacement (DHR)	82–93% [8,48]	0–8% [8,47]	0–25% [8,47]	0–3% [8,48]	3–6% [8,48]

**Table 2:** Summary of survival and complications “Lower Extremity”.

	Survival	Complications			
	(60 months)	Soft tissue failures (type 1)	Aseptic loosening (type 2)	Structural failure (type 3)	Infection (type 4)
Proximal Femoral Replacement (PFR)	55–90.7% [59,65,66,67,68]	4–33% [52,53]	0–11% [57]	0–3% [59,60,61] 19% [62]	6–19.5% [4,49,59,63]
Distal Femoral Replacement (DFR)	78% [74]	7–13.3% [72,73,74]	6.8–17.8% [8,72,74]	0–37.3% [4,72,73]	8.2–11.4% [8,74,78,82]
Proximal Tibial Replacement (PTR)	75% [74]	5.1–17.3% [73,74,86,87,89]	6–12% [74,86,87,88]	8.6–19% [74,80,86]	11.6–16.8% [4,73,82,86,88]
Total Femoral Replacement (TFR)	71–97% [71,91]	38–67% [67,91]	0–3.7% [8,67,71,91]	8.6–9% [67,71]	18.5% [71]
Combined Distal Femoral and Proximal Tibial Replacement (CFTR)	42% [94]	30.8% [94]	2.6% [94]	23.1% [94]	41% [94]

## Data Availability

No new data were created or analyzed in this study. Data sharing is not applicable to this article.
